# Effect of Hydrothermal Aging on Mechanical and Microstructural Properties of Zirconia Ceramics

**DOI:** 10.3390/nano15211669

**Published:** 2025-11-03

**Authors:** Çağlayan Sayla Çelik, Merve Çakırbay Tanış

**Affiliations:** Department of Prosthodontics, Faculty of Dentistry, Gazi University, Çankaya 06490, Ankara, Turkey

**Keywords:** monolithic zirconia, hydrothermal aging, mechanical properties, surface properties, elemental composition

## Abstract

The mechanical and microstructural properties of monolithic zirconia ceramics are significant factors for their long-term clinical performance. This study aims to investigate the effects of hydrothermal aging on these properties for the 3Y-TZP, 4Y-TZP, and 5Y-TZP formulations. Specimens were prepared from 3 different zirconia blocks: 3Y-TZP (HT), 4Y-TZP (ST), and 5Y-TZP (XT). Half of the specimens were aged in an autoclave (134 °C, 2 bar, 5 h) while the others remained as controls. Three-point flexural strength, Vickers hardness, and surface roughness tests, as well as XRD, AFM, and SEM/EDS analysis, were performed. The material type significantly affected the flexural strength, Vickers hardness, and surface roughness. Aging did not significantly affect the flexural strength or surface roughness but reduced the Vickers hardness in the 3Y-TZP sample. The 3Y-TZP and 5Y-TZP samples displayed the highest and lowest flexural strength, respectively. In the non-aged groups, 3Y-TZP and 5Y-TZP exhibited higher hardness than 4Y-TZP, and after aging, 3Y-TZP displayed the lowest hardness. Further, 5Y-TZP showed the highest surface roughness before and after aging. XRD revealed an increased monoclinic phase in the aged 3Y-TZP and 4Y-TZP. No monoclinic phase was observed in 5Y-TZP. According to AFM measurements, aging led to a smoother surface in 3Y-TZP but increased roughness in 4Y-TZP and 5Y-TZP. SEM/EDS revealed changes in the elemental compositions following aging. According to the results of this study, different material formulations affect the mechanical behavior and microstructural properties of monolithic zirconia ceramics. Further, hydrothermal aging displayed effects on the Vickers hardness and phase transformations.

## 1. Introduction

Recent years have seen an increased use of all-ceramic restorations rather than metal–ceramic ones due to their enhanced esthetic properties and biocompatibility. However, all-ceramic materials are physically weaker and more brittle, leading to the development of reinforced substructure materials to improve their mechanical properties. Among these, zirconium dioxide (zirconia) has gained popularity in dentistry due to its mechanical, chemical, physical, and esthetic advantages, in addition to its biocompatibility. Notably, zirconia is widely used in the fabrication of crown and bridge frameworks, orthodontic brackets, implant abutments and materials, telescopic attachments, and primary crowns [1].

Zirconia ceramics are used in the fabrication of veneered substructures or monolithic restorations. While veneering provides esthetic advantages, such systems lead to complications, such as porcelain chipping, and delamination, due to thermal expansion mismatches and weak interlayer bonding. This has led to the development of monolithic zirconia ceramics, including monoblock, full-contour, or anatomical zirconia [2,3,4]. These materials are characterized by their dense, nonporous structure; high biocompatibility; wear resistance, similar to that of natural enamel; and reduced wear on opposing dentition [4]. Monolithic zirconia restorations can be designed with minimal thickness (0.5 mm in posterior regions) with proper fracture resistance [5].

Structurally, pure zirconia exists in three crystallographic phases: monoclinic (at room temperature), tetragonal (at 1170 °C), and cubic (above 2370 °C). Upon cooling, the tetragonal phase transforms into the monoclinic one, leading to a 3–5% expansion in volume and subsequent microcrack formation. This transformation limits the clinical use of pure zirconia in dental restorations. To overcome this issue, stabilizing oxides such as Y_2_O_3_ are added to preserve the tetragonal phase at room temperature [6]. This stabilization also enables transformation toughening, a mechanism that enhances fracture resistance via the localized transformation of the tetragonal to the monoclinic phase under stress [7].

The first generation of monolithic zirconia was based on the 3 mol% yttria-stabilized zirconia (3Y-TZP) formulation, containing 3 mol% yttria and 0.25 wt% alumina, offering high strength but also leading to high opacity. To enhance the translucency, the alumina content was reduced to 0.05 wt%, and the yttria content was increased to 5 mol% (5Y-TZP) to promote the formation of the cubic phase [8]. However, the reduced mechanical properties of 5Y-TZP in multi-unit prostheses led to the development of 4Y-TZP, aiming to create a balance between mechanical strength and translucency with an yttria content of 4 mol% [9].

Zirconia ceramics are classified into four generations based on the yttria content: 3Y-TZP, modified 3Y-TZP (with a reduced alumina content), 5Y-TZP (with a high proportion of the cubic phase for improved esthetics), and 4Y-TZP (balancing strength and translucency) [10,11,12,13,14]. Additionally, multilayer zirconia blocks have been developed to better mimic the natural tooth structure and optimize optical properties [14].

Although Y-TZP have superior properties, these are influenced by the low-temperature (150–400 °C) degradation (LTD) phenomenon [15]. LTD is the transformation of zirconia from the tetragonal to the monoclinic phase (t ⟶ m) and happens under continuous hydrothermal stress by synovial fluid, water, or blood [16]. Water breaks zirconia molecules (ZrO_2_), forming zirconia oxide or breaks yttrium molecules into yttrium hydroxide (Y_2_O_3_) [17]. LTD is most evident between 200 and 300 °C [15]. The LTD of zirconia can cause microcracks and surface roughness. In addition, it can cause decreases in hardness, fracture toughness, and flexural strength, which may lead to deterioration [8].

The stability of dental zirconia is critical when subjected to aging and low-temperature degradation. Hydrothermal treatment in an autoclave is an accelerated method the simulates the long-term usage of the restoration in oral environment [16]. The clinical LTD of zirconia has been simulated in a steam autoclave at increased temperatures because the phase transformation is activated thermally and accelerated in the presence of water [18]. One hour of hydrothermal aging in an autoclave is equivalent to 3–4 years in vivo at 37 °C [8,18,19,20].

It is important to define the effect of LTD on monolithic zirconia ceramics to ensure long-term clinical success. Zirconia composition is also an important factor for clinical success, as zirconium dioxide restorations with different formulations may exhibit different behaviors. There are limited studies including all these parameters. This study aimed to define the effect of both hydrothermal aging and zirconia formulation on the mechanical and microstructural properties of zirconia ceramics. For this purpose, the flexural strength, Vickers surface hardness, surface roughness, surface topography, phase transformation, and elemental composition of 3 YTZ-P, 4 YTZ-P, and 5 YTZ-P zirconium dioxide ceramics were evaluated.

## 2. Materials and Methods

### 2.1. Preperation of the Specimens

Three pre-sintered monolithic zirconia ceramic blocks were used in this study: Vita-YZ-HT (Vita Zahnfabrik, Bad Säckingen, Germany), Vita-YZ-ST, and Vita-YZ-XT. Table 1 gives the composition and manufacturer details of the materials and material groups used in this study.

Specimens of appropriate dimensions were prepared from zirconia blocks for each test method using a low-speed cutting device (Microcut 201, Metkon Instruments Inc., Bursa, Turkey) with a 0.4 mm thick diamond disk under water cooling. A sintering shrinkage value of 20% was considered. A digital caliper was used to check the specimen dimensions. The specimens were ground and finished under water using 400, 600, 800, and 1200 grit silicon carbide (SiC) paper with a grinding and polishing machine (Metkon Gripo 2V, Metkon Instruments Inc.). Then, the specimens were placed in an ultrasonic cleaning device in distilled water for 15 min.

### 2.2. Sintering Procedure

The specimens were sintered using a sintering furnace (TABEO-1/S/ZIRKON-100, MIHM-VOGT GmbH & Co. KG, Stutensee-Blankenloch, Germany); the sintering parameters were determined according to the manufacturer’s instructions [21]. For the VITA YZ-HT, the sintering process was performed, starting from an initial temperature of 25 °C, with a heating rate of 17 °C/min, and held at 1450 °C for 120 min. For the VITA YZ-ST, sintering process started from 25 °C, with a heating rate of 8 °C/min, and was held at 1530 °C for 120 min. For the VITA YZ-XT, sintering started from 25 °C, with a heating rate of 4 °C/min, and was held at 1450 °C for 120 min. All sintering processes were carried out under controlled conditions, and the cooling phase continued down to 200 °C.

After sintering, the specimen dimensions were verified with a digital caliper, and specimens with dimensional differences were sized using the grinding and polishing machine and cleaned.

### 2.3. Hydrothermal Aging

The specimens were divided into two subgroups: half the specimens in each material group were subjected to hydrothermal aging, while the remaining ones served as the control.

Hydrothermal aging was performed to simulate the LTD of Y-TZP under humid conditions. The specimens were aged using an autoclave (CISA s.p.a., Pomezia, Rome, Italy) containing distilled water to create a saturated steam environment.

The autoclave was operated at 134 °C and 2 bar pressure for 5 h, following the accelerated aging conditions recommended by ISO 13356:2015 [22] for zirconia-based ceramics. During the process, the autoclave maintained a sinusoidal behavior of temperature and pressure at the beginning and end of the cycle, reaching and holding the target parameters (134 °C and 2.2 bar) during the main phase of the aging cycle.

After completion of the 5 h hydrothermal aging process, the samples were removed and allowed to cool naturally to room temperature.

### 2.4. Three-Point Flexural Test

A total of 72 specimens (n = 24 for each material group) with dimensions of 16 × 4 × 1.2 ± 0.2 mm were prepared in accordance with ISO 6872:2015 [23], and half the specimens in each material group were aged (n = 12).

Flexural testing was performed at room temperature (22 ± 1 °C) under dry conditions using a universal testing machine (Lloyd LRX, Lloyd Instruments, Fareham, UK). The specimens were placed on supports with a distance of 16 mm, and a load was applied at the midpoint of the specimens at a crosshead speed of 1 mm/min until fracture. The maximum load at fracture *P* (N) was recorded, and the flexural strength *σ* (MPa) was calculated using Equation (1):

*σ* = (3*PL*)/(2*bd*^2^) (1)

where *P* is the fracture load (N), *L* is the span length (16 mm), *b* is the specimen width (mm), and *d* is the specimen thickness (mm).

### 2.5. Vickers Hardness Test

Here, 72 specimens (n = 24 for each material group) with dimensions of 18 × 14 × 5 mm were prepared in accordance with ISO 6507-1:2018 [24], half of which were aged (n = 12).

The Vickers hardness was measured using a digital microhardness tester (HVS-1000, Aolong Xingdi Testing Equipment Co., Ltd., Shanghai, China). A 9.8 N load was applied for 15 s using a diamond indenter. The Vickers hardness *Hv* (GPa) was determined from five indentations per specimen using Equation (2):

*Hv* = (1.8544 × *P*)/*d*^2^ (2)

where *P* is the applied load (kg), and *d* is the average diagonal length (mm) of the indentation. The mean of five measurements was recorded for each specimen.

### 2.6. Surface Roughness Analysis

In the analysis of the surface roughness, 72 specimens (n = 24 for each material group) with dimensions of 15.5 ± 0.03 mm × 12.5 ± 0.03 mm × 1.2 ± 0.03 mm were prepared, half of which were aged (n = 12).

The surface roughness was measured using a contact profilometer (Mahr Perthometer M2, Göttingen, Germany). Three measurements were made along the central axis of each specimen, and the *Ra* (µm) value was determined to calculate the average of the three measurements.

### 2.7. X-Ray Diffraction (XRD)

Six zirconia specimens (n = 2 for each material group) with dimensions of 10 × 10 × 1 mm were prepared, half of which were aged (n = 1).

X-ray diffraction (XRD) analysis was performed using a diffractometer (Rigaku, Ultima IV, Tokyo, Japan) operating at 40 kV and 30 mA with a 2θ scanning range of 20–40°, a step size of 0.02°, and a scan speed of 0.6°/min. The diffraction patterns were then evaluated to identify the peak intensities corresponding to the monoclinic and tetragonal phases.

The monoclinic phase content (*Xm*) was calculated using the Garvie and Nicholson equation, as given in Equation (3):

*Xm* = [*Im*(−111) + *Im*(111)]/[*Im*(−111) + *Im*(111) + *It*(101)] (3)

where *Im*(–111) is the intensity at 2θ = 28.2° (monoclinic), *Im*(111) is the intensity at 2θ = 31.5° (monoclinic), and *It*(101) is the intensity at 2θ = 30.2° (tetragonal).

### 2.8. Atomic Force Microscopy (AFM)

Six specimens (n = 2 for each material group) with dimensions of 10 × 10 × 1 mm were prepared, half of which were aged (n = 1).

The surface topography of the specimens was analyzed using atomic force microscopy (AFM; Veeco MultiMode V, Santa Barbara, CA, USA) in contact mode. The scanning was performed at 2 Hz over areas of 10 × 10 µm and 5 × 5 µm.

### 2.9. SEM and Energy-Dispersive X-Ray Spectroscopy (EDS)

A total of 18 specimens (n = 6 for each material group) with dimensions of 10 × 10 × 1 mm were prepared, half of which were aged.

All specimens were sputter-coated with gold/palladium to enhance their surface conductivity. Their surface morphology and elemental composition were analyzed using scanning electron microscopy/energy-dispersive X-ray spectroscopy (SEM/EDS) at a magnification of 30,000× and an acceleration voltage of 30 kV. The weight percentages of Zr, Hf, Y, C, and O were then recorded.

### 2.10. Statistical Analysis

Statistical analyses were performed using SPSS software (version 26, IBM Corp., Armonk, NY, USA). The Kruskal–Wallis test was used to compare the materials and groups, and the Mann–Whitney U test was applied for the pairwise comparisons of significant variables. Independent samples *t*-tests were used to compare the aged and non-aged subgroups. A significance level of α = 0.05 was adopted for all statistical tests. In order to describe the reliability of the specimens, the Weibull modulus was calculated using the maximum likelihood method [25].

## 3. Results

### 3.1. Three-Point Flexural Test

The material formulations display a statistically significant effect on the three-point flexural strength (χ^2^ = 50.198, *p* < 0.5), while aging does not (t = 0.801, *p* > 0.5). A statistically significant difference was obtained between the groups (χ^2^ = 50.968, *p* < 0.05). The groups HT-non-aged, HT-aged, ST-non-aged and ST-aged showed statistically higher three-point flexural strength values than groups XT-non-aged and XT-aged (*p* < 0.05). Groups HT-non-aged and HT-aged showed statistically higher three-point flexural strength values than the ST-aged group (*p* < 0.05).

Within the non-aged groups, HT showed the highest Weibull modulus while the ST showed the lowest. Within the aged group, ST-aged showed the highest Weilbull modulus while the HT-aged showed the lowest.

### 3.2. Vickers Hardness Test

The material used (χ^2^ = 7.096, *p* < 0.5) and aging (t = 2.219, *p* > 0.5) display statistically significant effects on the Vickers hardness. Further, a statistically significant difference was observed among the groups (χ^2^ = 24.959, *p* < 0.05). A statistically significant difference was found between the Vickers hardness values of groups ST-aged and ST-aged and groups HT-non-aged, HT-aged, ST-non-aged and XT-non-aged (*p* < 0.05). Groups ST-aged and XT-aged showed statistically significantly higher Vickers hardness values than HT-non-aged, HT-aged, ST-non-aged and XT-non-aged groups. A statistically significant difference was found between the Vickers hardness values of HT-non-aged and XT-non-aged, and groups HT-aged and ST-non-aged groups (*p* < 0.05). The mean Vickers hardness values of HT-non-aged and XT-non-aged were found to be higher than those of groups HT-aged and ST-non-aged.

### 3.3. Surface Roughness Analysis

The material used has a statistically significant effect on the surface roughness (χ^2^ = 15.677, *p* < 0.005), while aging does not (t = 1.097, *p* > 0.5). A statistically significant difference was identified among the groups (χ^2^ = 17.395, *p* < 0.05). Groups XT-non-aged and XT-aged showed statistically higher surface roughness values than groups HT-non-aged, HT-aged, ST-non-aged, and ST-aged (*p* < 0.05). Groups HT-non-aged, HT-aged, ST-non-aged and ST-aged showed statistically similar results.

Table 2 presents the mean three-point flexural strength (MPa), Vickers hardness (GPa), Ra (µm) values, standard deviations, and statistical analysis of the groups.

Table 3 represents the Weilbull modulus and 95% Confidence Intervals (Cl) for 3-point flexural strength test.

### 3.4. XRD

**Figure 1:**
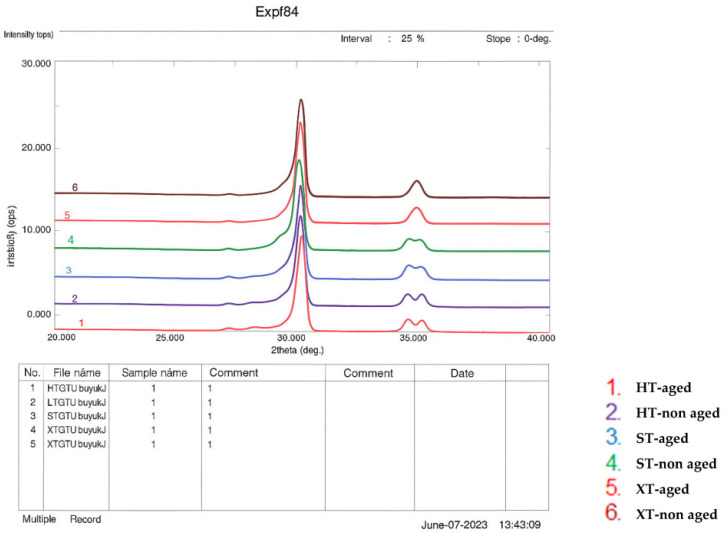
The XRD patterns of all groups.

Table 4 presents the monoclinic phase ratios (*Xm*) of each group, and Figure 1 shows the XRD patterns of all groups. Among the non-aged specimens, the highest *Xm* value occurs in group HT-non-aged, while no monoclinic phase was detected in group XT-non-aged. After aging, group HT-aged displays the highest *Xm*, with no monoclinic phase observed in group XT-aged.

### 3.5. AFM

**Figure 2:**
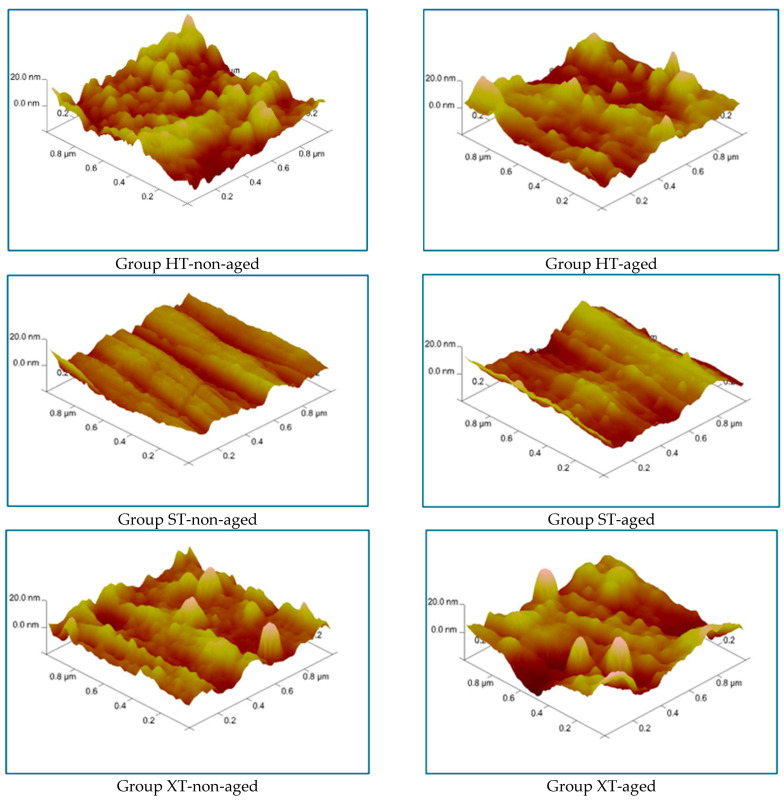
AFM images for each group.

Figure 2 presents the AFM images for each group. A rougher surface topography was observed in groups HT-non-aged and XT-non-aged, whereas group ST-non-aged exhibits a smoother and more homogeneous surface. The surface of group HT-non-aged is rougher compared to that of group XT-non-aged. The surfaces of groups HT-aged and XT-aged show rougher structures compared to group ST-aged. Additionally, the surface of group XT-aged is rougher than that of group HT-aged.

Distinct variations were identified between the surface characteristics of the non-aged and aged specimens. Group HT-aged exhibits a decrease in the surface roughness compared to group HT-non-aged, indicating a smoother and more homogeneous surface. Further, group ST-aged displays an increased surface roughness compared to group ST-non-aged. In group XT-aged, the surface roughness is higher than in group XT-non-aged, with noticeable porosity on the surface.

### 3.6. SEM/EDS

Table 5 presents the elemental compositions (C, O, Hf, Y, and Zr) of each group (wt%). Among the non-aged groups, the highest Zr content occurs in group HT-non-aged, with the lowest value in group XT-non-aged. Further, the highest and lowest Y contents were identified in groups XT-non-aged and HT-non-aged, respectively, and the highest and lowest O contents were observed in groups HT-non-aged and ST-non-aged, respectively. The aged groups display the following elemental compositions: Zr (highest, group XT-aged; lowest, group ST-aged), Y (highest, group XT-aged; lowest, group HT-aged), and O (highest, group HT-aged; lowest, group XT-aged).

## 4. Discussion

According to the results of this study, different formulations of monolithic zirconia ceramics affected the flexural strength, Vickers surface hardness, surface roughness, phase structure, surface topography, and elemental composition of the materials. Hydrothermal aging affected the Vickers surface hardness, phase structure, surface topography, and elemental composition of the specimens.

The highest flexural strength values were observed in the 3Y-TZP specimens, while the lowest occurred in the 5Y-TZP specimens, in agreement with previous studies [8,16,19,26,27,28,29,30]. The mechanical properties of zirconia are affected by its microstructure. Increasing the yttria content in 5Y-TZP causes the formation of a higher fraction of the cubic phase and reduces the amount of transformable tetragonal phase. Since the tetragonal phase contributes to transformation toughening, this reduction leads to lower flexural strength compared to 3Y-TZP [31].

Hydrothermal aging leads to a slight decrease in the flexural strength values, with no statistically significant effect. This agrees with some previous studies [16,19,26,30,32,33,34,35,36,37] reporting that aging had no effect on the flexural strength of monolithic zirconia. In contrast, Lümkemann et al. [8] found that hydrothermal aging increased the flexural strength of conventionally sintered 3Y-TZP but led to a decrease for conventionally sintered 5Y-TZP and preshaded high-speed sintered 4Y-TZP. In another study, Jerman et al. [38] found that hydrothermal aging increased the fracture strength of conventionally sintered 4Y-TZP but decreased that of high-speed sintered 4Y-TZP. According to Ha et al. [27] and Almansour et al. [39], aging leads to a statistically significant decrease in the flexural strength of conventionally sintered 3Y-TZP zirconia. Further, Kou et al. [20] evaluated the effect of aging on the flexural strength of two 5Y-TZP zirconia blocks (DD cubeX^2^, Dental Direct Materials, Germany ve Prettau Anterior, Zirkonzahn, Italy), reported a slight increase in the flexural strength of the Prettau Anterior specimens (with no statistically significant difference) and a statistically significant decrease in the flexural strength of the DD cubeX^2^ specimens. In another study, Zhang et al. [40] reported a statistically significant increase in the flexural strength of Y-TZP specimens with the aging time. The different results of these studies can be attributed to differences in the material brands, aging process, and sintering and testing parameters.

In the current study, the material type significantly influenced the Vickers surface hardness, though some previous studies have identified no statistically significant material-dependent difference [19,27,32]. Among the non-aged groups, 3Y-TZP and 5Y-TZP exhibited statistically similar Vickers surface hardness values, significantly higher than those of 4Y-TZP. Similar results have also been reported by Arcila et al. [41]. They have explained the lower Vickers hardness of 4 Y-TZP with the higher sintering temperature applied compared to 3 Y-TZP and 5 Y-TZP. Grambow et al. [42] reported that a decreasing sintering temperature resulted in a significant increase in Vickers hardness. Koo et al. [16] reported different results from this study. They found higher Vickers hardness values for non-aged 3 Y-TZP and 5 Y-TZP compared to 4 Y-TZP. The difference could be due to different material brands used. Among the aged specimens, 3Y-TZP exhibited the lowest values, while 4Y-TZP and 5Y-TZP showed statistically similar results. In contrast with this study, Alfahed et al. [31] and Toma et al. [43] reported higher surface hardness values for 4Y-TZP compared to 5Y-TZP, possibly due to the multilayered nature of the materials used and the surface treatments applied to the specimens.

Herein, aging significantly affected the Vickers hardness values. In contrast, certain previous studies [19,27,32,43,44,45] have reported that aging has no significant effect on the surface hardness of monolithic zirconia ceramics, which may be attributed to differences in the testing methods, material brands, or aging conditions. In the current study, aging reduced the Vickers hardness values of 3Y-TZP but increased those of 4Y-TZP and 5Y-TZP. Similarly, Kim et al. [35] reported a statistically significant reduction in the hardness values of 3Y-TZP specimens due to aging. In contrast, Rohr [44] and Alhotan [45] reported that aging did not affect the surface hardness of 3Y-TZP monolithic zirconia ceramics.

Profilometric analysis revealed that the material type significantly influenced the surface roughness. 5Y-TZP exhibited significantly higher roughness values compared to 3Y-TZP and 4Y-TZP, while no statistically significant difference was found between the surface roughness values of 3Y-TZP and 4Y-TZP. Peampring et al. [46] reported a higher degree of roughness in 3Y-TZP compared to 4Y-TZP and 5Y-TZP and increased surface roughness due to aging for 3 Y-TZP and 4 Y-TZP, in contrast with the findings of the current study. This could be due to differences in the brands of the zirconia blocks, aging parameters, and methods used between the two studies. Herein, aging did not significantly affect the surface roughness, in agreement with previous reports [20,40,47]; however, Koo et al. [16] reported an increase in the surface roughness following aging.

According to the XRD results, the tetragonal phase is predominant in all the materials before aging. The highest monoclinic phase content was observed in 3Y-TZP, both before and after hydrothermal aging. While some studies [9,18,19,28,30,36,46,47,48,49] have reported the absence of the monoclinic phase in non-aged 3Y-TZP, several studies [16,26,27,34,40,45] have noted its presence in 3Y-TZP before hydrothermal aging, supporting the findings of the current study. In addition, the monoclinic phase is present in 4Y-TZP before and after hydrothermal aging in the current study, in agreement with previous research [16,26,27]. Nevertheless, certain studies [9,28,34,46,48] have reported an absence of the monoclinic phase in non-aged 4Y-TZP. The monoclinic phase was not detected in 5Y-TZP before or after hydrothermal aging in the present study, in agreement with previous research [19,28,34,37,46,48]. On the other hand, other studies have reported the presence of the monoclinic phase in 5Y-TZP specimens before and after aging [20,26,27,50], in contrast with the findings of the current study. The differences between the XRD results of this study and other literature sources could be due to variations in material formulations, specimen preparation procedures, and aging times. Overall, hydrothermal aging led to an increase in the monoclinic phase content of 3Y-TZP and 4Y-TZP in the current research, aligning with previous studies [16,18,19,26,27,30,35,36,40,45,46,47,48,49,50].

AFM analysis revealed more prominent surface irregularities in the non-aged 3Y-TZP and 5Y-TZP specimens compared to the more homogeneous surface of 4Y-TZP. Hydrothermal aging improved the surface homogeneity of 3Y-TZP, in contrast with the findings of other studies [18,35,49]. Further, increased surface irregularities were observed in the 4Y-TZP and 5Y-TZP specimens, in agreement with previous studies [27,43].

According to the EDS analysis, 3Y-TZP exhibited the highest Zr and O contents among the non-aged specimens, while 5Y-TZP had the highest Y content; similar results have been reported by Arcila et al. [41]. In the aged specimens, 5Y-TZP showed the highest Zr and Y levels, while 3Y-TZP exhibited the highest O content. However, Koo [16] reported the highest Zr content for 4Y-TZP both before and after aging. In comparison with the current study, Ha et al. [27] reported similar Y and O results but different Zr ones, potentially due to the multilayered structure of the materials they employed. Alhotan [45] and Kim et al. [35] reported a decrease in the Y content of 3Y-TZP following aging; in contrast, the Y content increased due to hydrothermal aging in the current study. In another study, Kongkiatkamon et al. [37] reported that the contents of C and O in 5Y-TZP decreased slightly while those of Y, Zr, and Hf increased following aging, in agreement with this work.

## 5. Conclusions

Within the limitations of this study, the conclusions are as follows:

1. Different material formulations affected the mechanical behavior, surface roughness and microstructural properties of monolithic zirconia ceramics.
2. The highest flexural strength values were observed in 3 Y-TZP, while the lowest was observed in 5 Y-TZP. The use of 3 Y-TZP may be recommended in the presence of high chewing forces such as posterior restorations or bruxism.
3. Non-aged 3 Y-TZP and 5 Y-TZP showed highest and similar Vickers hardness values while aged 3 Y-TZP showed the lowest. According to these results, it can be concluded that the Vickers hardness of 3 Y-TZP is most affected by hydrothermal aging.
4. Hydrothermal aging displayed effects on the Vickers hardness, phase transformations, and elemental compositions of monolithic zirconia ceramics.

## Figures and Tables

**Table 1:** The composition and manufacturer details of the materials and material groups.

Material	Manufacturer	Material Composition	Material Groups
Vita-YZ-HT (3Y-TZP)	Vita Zahnfabrik, Bad Säckingen, Germany	90–95% ZrO_2_, 4–6% Y_2_O_3_, 1.5–2.5% HfO_2_, 0–0.3% Al_2_O_3_, 0–0.5% Er_2_O_3_, 0–0.3% Fe_2_O_3_	HT
Vita-YZ-ST (4Y-TZP)	Vita Zahnfabrik, Bad Säckingen, Germany	88–93% ZrO_2_, 6–8% Y_2_O_3_, 1.5–2.5% HfO_2_, 0–0.3% Al_2_O_3_, 0–0.5% Er_2_O_2_, 0–0.3% Fe_2_O_3_	ST
Vita-YZ-XT (5Y-TZP)	Vita Zahnfabrik, Bad Säckingen, Germany	86–91% ZrO_2_, 8–10% Y_2_O_3_, 1.5–2.5% HfO_2_, 0–0.3% Al_2_O_3_, 0–0.5% Er_2_O_3_, 0–0.3% Fe_2_O_3_	XT

ZrO_2_: zirconium dioxide, Y_2_O_3_: yttrium oxide HfO_2_: hafnium oxide, Al_2_O_3_: aluminum oxide, Er_2_O_3_: erbium oxide, Fe_2_O_3_: iron oxide.

**Table 2:** Mean three-point flexural strength (MPa), Vickers hardness (GPa), Ra (µm) values, standard deviations, and statistical analysis of the groups.

Groups	Flexural Strength (MPa) Mean ± SD	Vickers Hardness (GPa) Mean ± SD	Ra (µm) Mean ± SD
HT-non-aged	1192.1 ± 143.9 ^A^	1169.6 ± 46.8 ^B^	0.43 ± 0.14 ^A^
HT-aged	1112.8 ± 201.1 ^A^	1104.5 ± 44.2 ^A^	0.43 ± 0.17 ^A^
ST-non-aged	945.5 ± 398 ^A,B^	1129 ± 93.7 ^A^	0.52 ± 0.12 ^A^
ST-aged	850 ± 115.4 ^B^	1283.4 ± 145.5 ^C^	0.44 ± 0.14 ^A^
XT-non-aged	434.4 ± 100.6 ^C^	1170.2 ± 34.3 ^B^	0.7 ± 0.21 ^B^
XT-aged	401.5 ± 65.8 ^C^	1257.4 ± 153.3 ^C^	0.63 ± 0.19 ^B^

^A^, ^B^, ^C^: Letters indicate which groups differ significantly from each other. Different capital letters represent statistically significant differences between groups in each column.

**Table 3:** Weilbull modulus and 95% Cl for 3-point flexural strength test.

Groups	Weibull Modulus (m)	95% Cl
HT-non-aged	9.5 ^A^	(7.5; 11.4)
HT-aged	6.3 ^B^	(4.4; 8.3)
ST-non-aged	2.7 ^C^	(0.78; 4.7)
ST-aged	8.3 ^D^	(6.4; 10.3)
XT-non-aged	4.6 ^E^	(2.7; 6.6)
XT-aged	7.8 ^F^	(5.9; 9.8)

^A^, ^B^, ^C^, ^D^, ^E^, ^F^: Letters indicate which groups differ significantly from each other. Different capital letters represent statistically significant differences between groups in each column.

**Table 4:** Monoclinic phase ratios (Xm).

Groups	Monoclinic Phase Ratio (%)
HT-non-aged	2.13%
HT-aged	6.67%
ST-non-aged	1.47%
ST-aged	6.38%
XT-non-aged	0
XT-aged	0

**Table 5:** The elemental compositions of each group (wt%).

Groups	C	O	Hf	Y	Zr
HT-non-aged	13.95	11.62	2.28	6.64	65.5
HT-aged	12.76	12.47	2.34	8.75	63.68
ST-non-aged	13.93	10.76	2.29	9.68	63.14
ST-aged	11.38	12.24	3.27	9.65	63.47
XT-non-aged	15.21	11.51	2.9	9.85	60.53
XT-aged	10.59	10.68	2.84	11.89	63.99

C: carbon, O: oxygen, Hf: hafnium, Y: yttrium, Zr: zirconium.

## Data Availability

The data supporting reported results can be found, by writing to the corresponding author.
